# A DNA repair variant in *POLQ* (c.-1060A > G) is associated to hereditary breast cancer patients: a case–control study

**DOI:** 10.1186/1471-2407-14-850

**Published:** 2014-11-19

**Authors:** Ana Paula Carneiro Brandalize, Lavínia Schüler-Faccini, Jean-Sébastien Hoffmann, Maira Caleffi, Christophe Cazaux, Patricia Ashton-Prolla

**Affiliations:** Laboratory of Medical Genomics, Experimental Research Center, Hospital de Clínicas de Porto Alegre, Porto Alegre, Brazil; Department of Genetics, Federal University of Rio Grande do Sul, Porto Alegre, Brazil; Laboratory of Genomics, Proteomics and DNA Repair, University of Caxias do Sul, Caxias do Sul, Brazil; Instituto Nacional de Genética Médica Populacional, INAGEMP, Porto Alegre, Brazil; Equipe « Labellisée Ligue contre le Cancer 2013 » INSERM Unit 1037; CNRS ERL 5294, CRCT (Cancer Research Center of Toulouse), Toulouse Oncopole, France; University of Toulouse; UPS, F-31077 Toulouse, France; Hospital Moinhos de Vento, Porto Alegre, Brazil

**Keywords:** *POLQ*, DNA repair, Breast cancer, Translesional DNA polymerase, SNP

## Abstract

**Background:**

One of the hallmarks of cancer is the occurrence of high levels of chromosomal rearrangements as a result of inaccurate repair of double-strand breaks (DSB). Germline mutations in *BRCA* and *RAD51* genes, involved in DSB repair, are strongly associated with hereditary breast cancer. Pol θ, a translesional DNA polymerase specialized in the replication of damaged DNA, has been also shown to contribute to DNA synthesis associated to DSB repair. It is noteworthy that *POLQ* is highly expressed in breast tumors and this expression is able to predict patient outcome. The objective of this study was to analyze genetic variants related to *POLQ* as new population biomarkers of risk in hereditary (HBC) and sporadic (SBC) breast cancer.

**Methods:**

We analyzed through case–control study nine SNPs of *POLQ* in hereditary (HBC) and sporadic (SBC) breast cancer patients using Taqman Real Time PCR assays. Polymorphisms were systematically identified through the NCBI database and are located within exons or promoter regions. We recruited 204 breast cancer patients (101 SBC and 103 HBC) and 212 unaffected controls residing in Southern Brazil.

**Results:**

The rs581553 SNP located in the promoter region was strongly associated with HBC (c.-1060A > G; HBC GG = 15, Control TT = 8; OR = 5.67, CI95% = 2.26-14.20; p < 0.0001). Interestingly, 11 of 15 homozygotes for this polymorphism fulfilled criteria for Hereditary Breast and Ovarian Cancer (HBOC) syndrome. Furthermore, 12 of them developed bilateral breast cancer and one had a familial history of bilateral breast cancer. This polymorphism was also associated with bilateral breast cancer in 67 patients (OR = 9.86, CI95% = 3.81-25.54). There was no statistically significant difference of age at breast cancer diagnosis between SNP carriers and non-carriers.

**Conclusions:**

Considering that Pol θ is involved in DBS repair, our results suggest that this polymorphism may contribute to the etiology of HBC, particularly in patients with bilateral breast cancer.

## Background

Breast cancer is the major cause of cancer death among women worldwide. Although significant progress has been made to increase our knowledge on the mechanisms of carcinogenesis in the breast, the exact steps and contribution of each of these to breast cancer development remain elusive. Among the established risk factors for breast cancer are germline mutations in two highly penetrant genes: *BRCA1* and *BRCA2* [1, 2]. These mutations strongly increase breast cancer risk by disrupting homologous recombination repair (HRR) of DNA double strand breaks (DSBs) [3, 4]. Indeed, one of the hallmarks of cancer is the occurrence of high levels of chromosomal rearrangements as a result of inaccurate repair of DSBs [5]. Mutations in the *BRCA* genes and other genes encoding proteins involved in DSB repair (i.e. *RAD51*) are mostly associated with hereditary breast cancer and increase the genetic instability caused by DSBs [6–8].

DNA polymerase theta (Pol θ) is a recently identified translesional polymerase specialized in the replication of damaged DNA, which performs *in vitro* translesion synthesis at an AP sites and thymine glycol lesions. It is likely a major enzyme for such bypass in mammalian cells as it can perform both insertion in front of the damage and extension of the misincorporated nucleotide [9, 10]. It is an A-family polymerase composed of 2592 amino acids encoded by *POLQ* (3q13.31). Its function was first identified in *Drosophila*, where the mutant gene *MUS308* (ortholog of *POLQ*) was unable to survive to the chemical agents used to induce DNA breaks [11]. This polymerase possesses a unique structure with a conserved helicase domain at its N-terminal region, a polymerase domain at the C-terminus and a long central domain [12]. The lack of 3′-5′ exonuclease activity explains its low fidelity, generating substitutions at a frequency 10 to 100 times higher than observed with other family A polymerases [13].

Although the function of Polθ is not yet fully understood, several studies suggest that it may hold an important role in the maintenance of genome stability [14]. Pol θ has been proposed to be a backup DNA polymerase for base excision repair in chicken DT40 cells [15] and in nematodes [16, 17]. A role in the repair of DSB by performing DNA synthesis during alternative microhomology-mediated end-joining has been shown in *Drosophila* [18, 19] and this potential DNA repair function may explain why mouse Pol θ-deficient bone marrow and erythrocyte cells as well as human Pol θ-depleted tumor cells show increased sensitivity to ionizing radiation [20, 21]. It was also observed that Polθ possesses a polymerase activity in regions with DSBs [12]. *In vitro* studies in different organisms demonstrated that *POLQ* mutations resulted in abnormal DNA repair processes, decreased cell growth rates, arrest in G2 and increased chromosomal breaks and exchanges [9, 15, 16, 22].

In the context of cancer, the up-regulation of *POLQ* is observed in different tumor tissues, including lung, stomach, colon, breast, melanoma and oral squamous cell carcinomas [21, 23–27]. Recently, the expression of 13 human DNA polymerase genes was evaluated in breast carcinomas. Among these, *POLQ* showed the highest level of expression. Interestingly, patients with a more aggressive phenotype of breast cancer (triple negative), also had the highest levels of *POLQ* expression and lower survival (OR = 4.28; p = 0.0001), regardless of the levels of *CYCLIN E* and number of positive nodes [23]. These results were then confirmed in an independent cohort by Higgins et al. in 2010 (OR = 5.80; p = 0.001). In addition, fibroblasts transfected with a vector containing *POLQ* led to replicative stress and chromosomal instability [23].

Given the importance of DNA polymerase *POLQ* as genetic signature for the development and progression of breast cancer, the analysis of genetic variants in the *POLQ* gene represents a yet poor explored field of potential biomarkers in patients with breast cancer. Taken into account the possible involvement of *POLQ* in single and/or DSB repair, we hypothesized that variations in this DNA repair gene could drive the development of breast cancer. Here, we evaluated the possible contribution of nine SNPs in the *POLQ* gene to the development of both sporadic and hereditary breast cancer through a case–control approach.

## Methods

### Subjects

The subjects included in this study were divided into three groups: (1) Sporadic Breast Cancer (SBC), included women diagnosed with breast cancer above age 50 years who had no family history of breast cancer or other tumors; (2) Hereditary Breast Cancer (HBC), composed by women with a positive family history of breast cancer and other tumors and whose pedigrees met criteria for at least one of the hereditary breast cancer syndromes (HBOC – Hereditary Breast and Ovarian Cancer, HBCC – Hereditary Breast and Colon Cancer, SLF – Li-Fraumeni Syndrome or Li-Fraumeni like Syndrome) and excluding patients with known *BRCA* mutations; and (3) Patients without clinical evidence and/or suspected breast cancer participating in a mammographic screening program with normal (BIRADS 1 or 2) mammography scans within the last 12 months prior to sample collection. Along the study, a decision was made to include a fourth study group, and a cohort of 67 patients with bilateral breast cancer, regardless of age at diagnosis and family history of breast and other cancers was recruited. This study was performed independently of full *BRCA1, BRCA2, TP53* and *CHEK2* genotyping, based on the phenotype and clinical criteria for these syndromes. Thus, information on mutation status for high penetrance breast cancer genes is not available.

The study was approved by the Research Ethics Committee of Hospital de Clínicas de Porto Alegre (HCPA; protocol number 11–0328) and informed consent was obtained from all women before recruitment.

### SNP selection

Nine SNPs were systematically identified through the NCBI SNP database (http://www.ncbi.nlm.nih.gov/snp). SNPs were selected based on likelihood of affecting normal protein function (Table 1), association with an amino acid change in the protein (missense mutation) and location within exons. The most informative TagSNP and polymorphisms located in the putative transcription factor binding sites were identified using S NPinfo (http://www.snpinfo.niehs.nih.gov). Missense SNPs were included in our analysis due to their possible effect on protein function. The Polyphen prediction tool was used to predict a possible impact of this substitution. The Regulome DB was used to predict the effect of 5′UTR SNPs. SNPs with minor allele frequencies (MAF) described as less than 0.1 for European population databases were excluded.

**Table 1:** **Characteristics of selected** ***POLQ*** **SNPs**

SNP ID	Region	Protein domain	Mutation type	Aminoacid change	Possible functional effect
rs587553	-1060				Putative TFBS*
rs13065220	-190				Putative TFBS*
rs3806614	-323				Putative TFBS*
rs11713643	intron 1		TagSNP		Tags 23 of 56 SNPs listed at SNP info
rs41545723	Exon 4	helicase	missense	Leu197Arg	possibly damaging**
rs61757736	Exon 6	helicase	missense	Ser305Ala	possibly damaging**
rs55748151	Exon 6	helicase	missense	Val310Gly	possibly damaging**
rs3218651	Exon 16	central	missense	His1201Arg	possibly damaging**
rs1381057	Exon 28	polymerase	missense	Gly2513Arg	possibly damaging**

*TFBS = Transcription Binding Factor Site; Functional effect predicted by regulome DB.

**Funciontal effect predicted by Polyphen.

### Genotyping

Peripheral blood samples collected in EDTA tubes were subjected to DNA extraction using the GE extraction kit (GE Healthcare Lifesciences BR). TaqMan assays were used for SNP genotyping. Genotypes were determined by the TaqMan probes C_88490787-10, C_86270772-10, C_88490786, C_919228-10, C_8248307-20, C_31746782-10, C_3100675-10 and C_61757736. Two custom assays were specially developed, one for the Tag SNP and another for rs11713643 (Applied Biosystems). Real-time PCR reactions were performed in 48-well plates. Briefly, each reaction contained 20ng of genomic DNA, 6.25 μl of 2x MasterMix Genotyping TaqMan (Applied Biosystems), 0.31 μl of probes specific for each polymorphism (40x) and 4.94 μl of DNase-free water. A StepOne PCR Real-Time System was used for all reactions, with an initial cycle of 10 minutes at 95°C, followed by 45 cycles of 15 seconds at 92°C and 1 minute at 60°C.

### Statistical analyses

The Chi-square test was used to assess deviation from Hardy-Weinberg equilibrium and to compare allele and genotypic frequencies between cases and control groups. For each statistically significant association, an unconditional binary logistic regression model was fitted to calculate the odds ratios (ORs) and 95% confidence intervals (CIs). Statistical analyses were performed using SPSS software version 18.0.

## Results

A total of 103 HBC and 101 SBC patients were included in the case groups, and their clinical characteristics are summarized in Table 2. The control group included 212 women unaffected by breast cancer whose mean age was 56 years (SD = 5.8 years).

**Table 2:** **Clinical characteristics of HBC and SBC groups**

Characteristic	HBC			SBC
	HBOC	HBCC	Other*	
Number of patients, n (%)	77 (74.8)	4 (3.9)	22 (21.4)	101 (100)
Age at diagnosis, mean (sd)	45.75 (11.8)	47.66 (3.2)	41.1 (10.4)	56.9 (5.2)
Age at diagnosis >50, n (%)	25 (32.5)	0	6 (27.3)	101 (100)
Bilateral breast cancer, n (%)	50 (74.6)	3 (4.5)	5 (7.5)	9 (13.4)
Synchronic	23 (47.8)	2 (66.7)	-	2 (22.2)
Metachronic	26 (50.0)	0	-	7 (77.8)
Missing	1 (2.2)	1 (33.3)	5 (100)	0

*Other: Li-Fraumeni, Li-Fraumeni like or HBCC syndromes.

The observed distribution of *POLQ* genotypes and allele frequencies in all groups, as well as their respective frequencies in 1000 Genomes database are shown in Table 3. Since the geographic region of patient recruitment is extensively colonized by Europeans [28], we used the expected allelic frequencies published in 1000 Genomes for euro-descendent populations as reference to minimize effects of population admixture in the analysis. Three of the nine SNPs studied (rs61757736, rs41545723, rs55748151) were not found in any of the groups. The allelic distribution for the other polymorphisms were in Hardy-Weinberg equilibrium in all groups, and the observed allele frequencies in controls are in agreement with those described for euro-descendants in the 1000 Genomes database. Overall, the *POLQ* genotype frequencies were equally distributed in SBC and controls with the exception of rs581553 located in the promoter region of the gene, which was strongly associated with an increased risk for HBC (c.-1060A > G; HBC GG = 15, Control GG = 8; OR = 5.67, CI95% = 2.26-14.20; p < 0.0001). Specifically rs581553 allele G was associated with HBC as well (OR = 2.33, CI95% = 1.57-3.47; p < 0.0001). Surprisingly, presence of the polymorphic allele c.3602G, localized in the central domain of Pol θ was associated with a protective effect for breast cancer in the SBC group (OR = 0.59, CI95% = 0.36-0.96; p < 0.027).

**Table 3:** **Genotypic and allelic frequency of selected** ***POLQ*** **polymorphisms in HBC, SBC and control groups**

SNP ID	Genotype	HBC		Control		p	SBC		Control		p	1000 Genomes
		n = 103	%	n = 212	%		n = 101	%	n = 212	%		
	AA	48	46.60	110	51.89		44	43.56	110	51.89		
rs1381057	AG	46	44.66	86	40.57		49	48.51	86	40.57		
c.7538A > G	GG	9	8.74	16	7.55	0.675	8	7.92	16	7.55	0.372	
	G	64	31.07	118	27.83	0.401	65	32.18	118	27.83	0.301	28
	AA	47	45.63	142	66.98		59	58.42	142	66.98		
rs587553	AG	41	39.81	62	29.25		34	33.66	62	29.25		
c.-1060A > G	GG	15	14.56	8	3.77	0.0001^a^	8	7.92	8	3.77	0.172	
	G	71	34.47	78	18.40	0.0001^b^	50	24.75	78	18.40	0.072	19
	AA	44	42.72	108	50.94		44	43.56	108	50.94		
rs13065220	GA	49	47.57	89	41.98		49	48.51	89	41.98		
c.-190G > A	GG	10	9.71	15	7.08	0.355	8	7.92	15	7.08	0.473	
	G	69	33.49	119	28.07	0.165	65	32.18	119	28.07	0.303	28
	CC	36	34.95	97	45.75		39	38.61	97	45.75		
rs3806614	CT	54	52.43	88	41.51		48	47.52	88	41.51		
c.-323C > T	TT	13	12.62	27	12.74	0.153	14	13.86	27	12.74	0.487	
	T	80	38.84	142	33.49	0.213	76	37.62	142	33.49	0.324	33
	AA	70	67.96	132	62.26		76	75.25	132	62.26		
rs3218651	AG	28	27.18	72	33.96		23	22.77	72	33.96		
c.3602A > G	GG	5	4.85	8	3.77	0.461	2	1.98	8	3.77	0.073	
	G	38	18.44	88	20.75	0.526	27	13.37	88	20.75	0.027^c^	28
tag SNP	TT	36	34.95	91	42.92		48	47.52	91	42.92		
rs11713643	TC	51	49.51	93	43.87		38	37.62	93	43.87		
c.2730T > C	CC	16	15.53	28	13.21	0.398	15	14.85	28	13.21	0.576	
	C	83	40.29	149	35.14	0.218	68	33.66	149	35.14	0.788	36

^a^χ^2^ test, p < 0.0001, OR = 5.67, CI95% = 2.26-14.20.

^b^χ^2^ test, p < 0.0001, OR = 2.33, CI95% = 1.57-3.47.

^c^χ^2^ test, p < 0.027, OR = 0.59, CI95% = 0.36-0.96.

Based on the strong association identified between *POLQ* c.-1060A > G (rs581553) and HBC, we carefully reviewed the clinical features of the patients with and without the polymorphic genotype c.-1060GG, in an attempt to find a clue for its possible functional role. Interestingly, 11 of the 15 GG homozygotes fulfilled criteria for HBOC syndrome. Furthermore, 12 of them had been diagnosed with bilateral breast cancer and an additional patient reported a familial history of bilateral breast cancer. To verify whether bilateral breast cancer was associated with this polymorphism we analysed rs581553 in an additional group of 67 patients with bilateral breast cancer and a positive family history of either breast or breast and ovarian cancer. When the genotypic frequencies were compared, a statistically significant association remained (Bilateral Breast Cancer GG = 15; OR = 9.86, CI95% = 3.81-25.54; p < 0.0001), as shown in Table 4. We did not observe an association between presence of G allele and synchronous or metachronous bilateral breast cancer (p = 0.887; data not shown). Finally, we hypothesized that the presence of this polymorphism could facilitate or promote occurrence of early onset breast cancer and analysed a group of patients with early-onset breast cancer (age at diagnosis <40 years) for a potential association of this SNP. No statistically significant association was found (p = 0.744; data not shown).

**Table 4:** **Genotypic frequency of** ***POLQ*** **c.-1060A > G in bilateral breast cancer patients and controls**

SNP ID	Genotype	Bilateral		Control		p
		n = 67	%	n = 212	%	
rs587553	AA	27	40.30	142	66.98	
	AG	25	37.31	62	29.25	
	GG	15	22.39	8	3.77	0.0001*

*χ^2^ test, p < 0.0001, OR = 9.86, CI95% = 3.81-25.54.

## Discussion

Deficient DNA repair is one of the most prominent risk factors for tumor development and genetic variations in DNA repair genes have been shown to play an important role in the carcinogenesis of breast cancer. Several genes are responsible for performing DSB DNA repair, and mutations in *BRCA* genes explain around 30% of all hereditary breast cancers. Some studies indicate that the occurrence of breast cancer in families with no *BRCA* mutations, as our HBC group, could be explained by the existence of low penetrance polymorphisms in several repair genes [1, 29]. Accumulating evidence has suggested a potential role for *POLQ* in mammalian DSB repair. *POLQ* deficient mice show increased sensitivity to low doses of bleomycin in their bone marrow cells [20], and the same sensitivity has been observed in human tumor cells exposed to ionizing radiation [21]. Furthermore, knockdown of *POLQ* in mouse lymphoma cells increases their sensitivity to etoposide [30]. All of these agents are established mutagens that produce DNA DSB. Additional evidence supporting a role of *POLQ* in maintaining genomic stability comes from studies in Chaos-1 mice, where a missense mutation at position 1932 of the *POLQ* gene, is associated with high levels of micronuclei, and increased levels of chromosome breakage. At the same time, *POLQ* seems to have a unique role in DSB repair that complements the recombination machinery regulated by *ATM* in HRR since *POLQ* knockout mice display enhanced chromosome instability in ATM-deficient cells [31]. The identification of the human *POLQ* ortholog in mus308/*Drosophila* as well as in mus301/spn-C, which is involved in meiotic DSBs repair and checkpoint activation [32], reinforces the potential involvement of *POLQ* in HRR. *POLQ* seems to play an additional role in DSB repair utilizing as substrate DNA by the incorporation of random nucleotide sequences [33]. Finally, the tolerance to DSB is not uniquely dependent on the polymerase activity of Pol θ. The protein’s helicase domain is likely involved [34]. In Drosophila, *POLQ* acts in this sites where its helicase activity unwinds short stretches of DSBs to expose pre-existing microhomologous sequences that are used to align the broken ends to provide a template for pol θ polymerase activity [19]. Definitely, the mechanism in which Pol θ coordinates its polymerase and helicase activities to participate in repair of different types of DNA lesions remains to be determined.

Here we showed that a specific SNP in the promoter region of *POLQ* is associated the phenotype of hereditary breast and ovarian cancer syndrome and/or with bilateral breast cancer, but not sporadic breast cancer. To the best of our knowledge this study is the first to demonstrate an association between a *POLQ* SNP and increased risk for multiple primary breast tumors. In 2008, a germline frameshift mutation, expected to disrupt the polymerase activity, was identified in one patient with a personal and familial history of breast cancer. The authors also described five missense variants in *POLQ* in other 38 women with SBC, but none of these variants seemed to affect gene function [2]. Recently, the effects of 11 *POLQ* SNPs were studied in a population-based series of 783 Swedish breast cancer patients, but no association was observed [35]. In agreement with our data, these studies did not find a strong association between SBC and *POLQ* variants probably because different mechanisms influence chromosomal instability and carcinogenesis in sporadic and hereditary breast cancers. The components of genetic susceptibility in SBC, where a highly penetrant germline mutation in a cancer predisposition gene is not identified, are still poorly understood and the environmental aetiological factors definitively contribute to increase the risk. In addition, this and other previous studies of *POLQ* variants are limited in sample size to identify significant associations of lower penetrance variants.

In favour of a deleterious effect of *POLQ* c.-1060GG, its frequency in controls was relatively low (around 3%) as compared to the frequency in patients with HBC (around 15%). It is also interesting to note that the majority (73.3%) of patients harbouring the homozygous mutant genotype in the HBC group had a personal and family history consistent with HBOC. The development of multiple primary breast tumors (bilateral breast tumor) can be associated to disrupted *POLQ* expression that may be involved in DSB repair. In hereditary cancers, the presence of chromosomal instability is linked to germline mutations in genes associated with DNA DSB or interstrand cross-links repair [36]. Such mutations have been previously associated with bilateral breast cancer in several populations [37, 38].

According to Regulome DB database (http://regulome.stanford.edu/index), *POLQ* c.-1060A > G is located in a putative transcription binding factor site of Ying Yang1 protein, encoded by the *YY1* gene. This protein is a ubiquitous, conserved, multifunctional zinc-finger transcription factor that regulates initiation, activation, or repression of transcription from a variety of genes required for cell growth, development, differentiation and tumor suppression [39–42]. The DNA-binding activity of *YY1* increases dramatically early in S phase [43], where *POLQ* would be present to perform its translesion or repair activity. It is notyet known whether *YY1* negatively or positively regulates the expression of *POLQ*. However, the Yin Yang 1 protein positively regulates *BRCA1* and inhibits breast cancer formation [41]. On the other hand, *YY1*-deficient spermatocytes show univalent formation, increased aneuploidy, and pachytene cell death, which are likely due to defects in DNA repair [44]. Thus, we hypothesize that the presence of *POLQ* c.-1060A > G SNP prevents Ying Yang-1 binding to its transcription binding site, disrupting *POLQ* expression. This in turn disturbs or diminishes DSB repair, leading to a phenotype of increased chromosomal instability, as observed in *BRCA*-deficient and other hereditary breast cancer phenotypes.

## Conclusions

Our data contributes to previous evidence suggesting that downregulation or absence of *POLQ* expression leads to inaccurate DSB repair. Thus *POLQ* could be considered as an important player in breast carcinogenesis, acting in this context as a tumor suppressor gene due to its important role in DNA repair. Further analysis to explain the functional consequences of *POLQ* c.-1060GG on *YY1-*mediated *POLQ* expression and on breast cancer progression are warranted. The whole sequencing of *POLQ* gene and its untranslated regions would also be fundamental to determine whether these inherited genetic variations can predispose women to breast cancer, and particularly to bilateral breast cancer.

## Abbreviations

DSB: Double strand breaks
HBC: Hereditary breast cancer
HBOC: Hereditary breast and ovarian cancer syndrome
HBCC: Hereditary breast and colon cancer syndrome
HCPA: Clinical hospital of porto alegre
CI95%: Confidence interval
HRR: Homologous recombination repair
MAF: Minor allele frequencies
OR: Odds ratio
PCR: Polymerase chain reaction
SBC: Sporadic breast cancer
SLF: Li-Fraumeni syndrome or li-fraumeni like syndrome
SNP: Single nucleotide polymorphism
UTR: Untranslated region
SD: Standard deviation.
